# Minimally invasive mesh salvaging technique on treatment of hernia mesh infection: A case series

**DOI:** 10.1016/j.ijscr.2020.04.055

**Published:** 2020-05-11

**Authors:** Arnolds Jezupovs

**Affiliations:** a Clinic of Surgical Infections, Riga East University Hospital, Riga, Latvia; b Faculty of Medicine, University of Latvia, Riga, Latvia

**Keywords:** Hernia, Mesh infection, Mesh salvage, Irrigation, Povidone-iodine, Case series

## Abstract

**Introduction:**

The aim of the presentation is to demonstrate a new conservative approach for the salvage treatment of the infected hernia mesh.

**Methods:**

Three patients with polypropylene mesh infection following an open umbilical and incisional hernia repair performed by placing the mesh in the onlay position are presented. The infection manifested itself with a systemic inflammatory response and an infected fluid collection surrounding the mesh. All patients were treated conservatively by a minimally invasive technique according to the stepwise protocol.

**Results:**

An effective, rapid response to the conservative treatment was observed. A total of five to six procedures were necessary in a period of up to twenty days. Mesh salvage was achieved, and the long-term outcome demonstrates neither the recurrence of infection nor hernia.

An enclosed space at the infection site is the main precondition for providing a sufficient concentration of the antiseptic for a long enough time to act effectively at the infection site. The fast killing effect on the mesh infection and a good long-term outcome in all presented patients prove that this technique is superior to the the current techniques in appropriately selected patients from the point of treatment duration.

**Conclusion:**

An effective mesh salvaging conservative treatment is possible when the infection manifests itself as an infected fluid collection surrounding the monofilament, large-pore polypropylene mesh and should be the first-line option. Open surgical approach should be reserved as a second-line option if the first fails.

## Highlights

• Infected monofilament, large-pore polypropylene hernia mesh can be salvaged.
• Instillation of topical antiseptics provides recovery from hernia mesh infection.
• An enclosed space provides a sufficient concentration of the antiseptic.
• Povidone-iodine is effective on hernia mesh infection.

## 1 Introduction

It is widely accepted that a prosthetic mesh implant demonstrates a clear benefit in reducing hernia recurrence compared to suture repair alone in the abdominal wall hernia repair [1].

However, mesh infection is a devastating complication harmfully affecting all involved and can result in mesh explantation, beginning the hernia repair cycle again.

The current mesh salvaging approaches consist of either open techniques [[2], [3], [4]] or percutaneous aspiration followed by continuous drainage with short-acting antibiotic flushes [5,6]. Both approaches are long-lasting. Furthermore, the open method is expensive too if negative pressure wound therapy is applied.

The current presentation demonstrates a new, simple and cost-effective conservative approach for the salvage treatment of the infected mesh graft, consisting of intermittent aspirations and irrigations followed by instillation of long time exposure topical antiseptics.

## 2 Methods

This work has been reported in line with the PROCESS criteria [7].

Three patients with implant infection following an open umbilical and incisional hernia repair performed by placing the mesh in the onlay position are presented. Monofilament, large-pore, mid-weight polypropylene mesh was utilized in all patients. A 24 Fr silicon drain was put in the subcutaneous space over the hernia mesh in Cases 1–2, and a 15 Fr silicon drain in Case 3.

The infection manifested itself with a systemic inflammatory response and an infected fluid collection surrounding the mesh. All patients were treated conservatively by a minimally invasive technique according to the stepwise protocol (Table 1) in a single institution by the author of the article.

**Table 1:** Practical stepwise protocol for treatment of infected fluid collections surrounding mesh.

Step	Action	Effect
1	A blind percutaneous puncture of the abscess cavity and aspiration of the content by a 14–18 gauge needle.	Pus is evacuated.
2	Irrigation of the abscess cavity via the same needle with an aqueous antiseptic agent for topical use in the same volume as aspirated in Step 1. This is repeated many times till the return is clear.	Residual pus and debris are evacuated. Reduction of the bacterial load.
3	Refilling of the abscess cavity with an aqueous antiseptic agent for topical use with the same volume as aspirated in Step 1 and leaving it at the infection site till the next aspiration.	Provision of a deadly concentration of antiseptic agent at the entire infection site.
4	Every following cycle of the procedure is repeated in 6–24 h for 2–4 days.	Provision of a deadly concentration of antiseptic agent at the entire infection site for a sufficiently long acting time to eliminate the infection.
5*	A few puncture aspirations might be performed during the next few weeks for controlling purpose.	Infection control and evacuation of seroma. No need to perform irrigation if aspirate is clear.

* Not mandatory to perform if ultrasound monitoring is available.

## 3 Results

### 3.1 Case 1

A 45-year-old female was admitted to hospital for an elective large incisional hernia repair. She had a history of open cholecystectomy through an upper midline laparotomy due to acute cholecystitis two years ago. The postoperative course was complicated due to a deep wound infection and finally resulted in the forming of a large ventral hernia at the incision site. The patient had a history of diabetes mellitus treated with insulin, abuse of alcohol and smoking.

Clinical examination revealed no signs of infection at the hernia site. There were no restrictions for an elective surgery.

Open incisional hernia repair was performed on September 28, 2006 by mesh sized 225 cm^2^. Intra-abdominal adhesiolysis was done simultaneously because of hard adhesions between the intestinal loops and the abdominal wall. Any spots of residual infection were not found during hernia repair. The patient received 2 g of Cefazolin 30 min prior to incision. Antibiotics were not administered after surgery.

The immediate postoperative course was uncomplicated. Bacterial growth on the swabs taken from the patient's skin before the incision and the mesh surface before the wound closure was not confirmed. The subcutaneous drain was removed and the patient was discharged on the 4th post-operative day.

A routine follow-up visit at an outpatient office two months after the surgery revealed no signs of complications.

The patient presented at the outpatient office on an emergency basis one month later with fever and abdominal pain at the site of the scar. The patient had been well until five days before this presentation when fever, chills and abdominal pain developed. Despite the oral use of Ibumetin, her symptoms did not regress.

Examination revealed a typical clinical presentation of abscess on the distal part of the scar 5.0 × 6.0 cm in size approximately. The patient`s temperature was 38.8 °C.

As there was no possibility to perform urgent surgery and to relieve the patient's suffering, a blind percutaneous puncture of the abscess was performed by an 18 gauge needle.

80 mL of grey foul-smelling pus was aspirated. Then 10 mL of a 3% hydrogen peroxide solution was injected into the abscess cavity through the same needle and re-aspirated. Further irrigation was continued by 80 mL of a 10 % sodium chloride solution many times by turn till the return was clear, and the needle was removed after refilling the abscess cavity by 80 mL of a 0.1 % Furagin solution.

The patient was instructed to arrive at hospital for open surgery in the next morning.

The following morning when the patient was examined in hospital, her condition had improved beyond all expectations in eleven hours’ time. The temperature had dropped to 37.1 °C. Pain was discontinued, and the patient suffered only from mild discomfort at the infection site. Local presentation of infection signs was also reduced convincingly. 40 mL of bloody turbid exudate was aspirated on puncture and sent for bacterial growth examination. The abscess cavity was irrigated by 40 mL of normal saline and 40 mL of a 1% povidone-iodine aqueous solution alternately many times in turns till the return was clear, and 40 mL of a 1% povidone-iodine aqueous solution was left in the abscess cavity up to the next puncture time.

A 1% povidone-iodine aqueous solution was prepared by a 1:9 dilution of full strength (10 %) povidone-iodine aqueous solution with a normal saline solution giving 0.1 % available iodine in a diluted form.

Based on the clinical improvement, a decision was made to postpone surgical treatment and to continue a conservative one on an outpatient basis. A ciprofloxacin course was administered perorally for 10 days.

The patient was under close supervision of the author for the next eight days. The treatment procedures were continued according to the stepwise protocol (Table 1). By the 5th day after the treatment had begun, the aspirate become seroma-like. Bacterial examination of this fluid collection did not confirm bacterial growth anymore. The treatment was stopped on the 10th day after the first puncture because the aspirate volume had decreased to 25 mL and continued to be clear on appearance.

At 12 months after completing the treatment the patient remained clinically free from infection and hernia recurrence. The patient missed the follow-up later. An attempt to find her through the assistance of the national register service in 2019 resulted in the confirmation of the patient`s death in 2010 from an unknown reason.

### 3.2 Case 2

A 61-year-old man was presented with a large incisional ventral and left-sided groin hernia. He had a history of open cholecystectomy through a vertical midline laparotomy due to acute gangrenous cholecystitis two years ago. The patient was re-operated on due to evisceration on the next day. The postoperative course was complicated by a deep wound infection and secondary wound healing subsequently. Incisional hernia formed at the incision site later. The patient had a history of heavy smoking and controlled chronic obstructive pulmonary disease.

Open simultaneous repair of both hernias was done on April 23, 2007. A mesh sized 225 cm^2^ was applied for the incisional hernia, and 66 cm^2^ for the groin hernia repair performed by the Lichtenstein method. Starting from the first postoperative day, the patient`s temperature was elevated and hit a high of 38.0 °C in the evenings. Therapy with Gentamicin 240 mg was administered parenterally once daily, starting from postoperative day one. A brownish turbid exude through the drain appeared on the second postoperative day; besides, the drain fell out late in the same evening. Sutures were removed from the inguinal wound on the 4th day postoperatively because of infection signs, but the patient`s temperature did not normalize. A blind puncture of the incisional hernia site was done on the 7th postoperative day in order to examine the fluctuating mass. Brownish pus was aspirated. Bacterial testing confirmed the same microorganism growing as in the groin. Treatment according to the stepwise protocol (Table 1) was initiated. A gradual improvement was observed. By the 6th day after the first puncture, the aspirate looked like seroma. The patient was discharged from the hospital on the 20th day postoperatively. The incisional hernia wound had healed by primary intention, and the open inguinal hernia wound caused by superficial infection healed spontaneously in six weeks.

The follow-up at 12 years after the treatment did not reveal signs of infection or both hernia recurrences.

### 3.3 Case 3

A 60-year-old female revisited the hospital following ventral hernia repair with a four-day history of fewer and abdominal pain at the surgery site. Irreducible umbilical hernia had been repaired seven days ago (on September 30, 2009) by mesh sized 96 cm^2^. The Omentum majus was partially resected during the procedure because of the inability to move it back into the abdominal cavity. The patient`s comorbidities consisted of severe adiposity and controlled bronchial asthma. The early postoperative course was uneventful. The subcutaneous drain was utilized on day two postoperatively, and she was discharged from the hospital the same day. The next day, the patient`s body temperature increased to 38.8 °C and pain at the surgery site became more prominent. She started oral antibacterial therapy with 1.2 g of amoxicillin/clavulanic acid three times daily but without improvement.

On examination, a tender, warm erythema was found overlying the incision. Fluctuation just above the sutures was revealed, but discharge from the wound was absent. The patient`s temperature was 37.8 °C.

A blind percutaneous puncture of the fluctuant area was performed by a 14 gauge needle, and a turbid brownish fluid was aspirated in which bacterial testing confirmed heavy bacterial growth. The patient was put on a five-day ciprofloxacin course perorally and proceeded to the stepwise treatment protocol (Table 1) which was stopped in three days because of the elimination of inflammatory signs. The patient was seen again in seven days for suture removal and control examination. The wound had healed by primary intention. Tissue hardening around the scar was observed. An attempt to control the cavity by blind puncture resulted in not obtaining any content. The last follow-up 10 years after the treatment confirmed that the patient continues to be free from infection and hernia recurrence.

The patients` mesh infection presentation and treatment details are seen in Table 2.

**Table 2:** Mesh infection presentation and treatment summary.

Patient	Infection confirmation after hernia repair	Infection duration before the first puncture	Volume of pus aspirated the first time (a causative microorganism)	Applied aqueous topical agents	Total number of punctures/instillations (time between the first and last puncture)
Case 1 patient	in 92 days	5 days	**80 m**l (*Enterobacter cloacae)*	Hydrogen peroxide 3% Normal saline NaCl 10 % Furagin 0,1%	6/5 (10 days)
Case 2 patient	in 7 days	7 days	80 mL (*Staphylococcus capitis)*	Hydrogen peroxide 3% Normal saline Povidone - iodine 1%	6/4 (20 days)
Case 3 patient	in 4 days	2 days	40 mL (*Pseudomona diminuta)*		5/4 (9 days)

## 4 Discussion

The current study demonstrates a successful implant salvage treatment in patients with polypropylene mesh infection by a minimally invasive approach. To the author`s knowledge, such approach has never been described.

The presented stepwise technique has some important differences which distinguish it from similar studies [5,6].

The drainage of the infected fluid collection and irrigation were managed intermittently through blind punctures followed by instillation of antiseptics, thus the presence of topical antibacterial agents at the infection site was provided on an ongoing basis over the whole infected surface for many hours. Finally, topical antiseptics were applied instead of antibiotics.

The fast killing effect on the mesh infection and a successful long-term outcome in all study patients prove that this simple, economically beneficial intermittent aspiration and irrigation technique is superior to open or continuous percutaneous drainage with short-acting antibiotic flushes in appropriately selected patients from the point of treatment duration.

A monofilament, large-pore polypropylene mesh has the greatest chance to be salvaged [8,9]. Such mesh was applied in the presented patients.

It is common for surgeons to make an incision when an abscess is present. The efficacy of the presented technique was discovered accidentally due to the inability to make an urgent incision.

An enclosed space at the infection site is the main precondition for following the aforementioned stepwise protocol. Only a hermetic space ensures a constant concentration of the antimicrobial agent at the infection site for a sufficient time period to act effectively. This method is ineffective in all cases of limited leak-tightness. Thus it is not useful after spontaneous or surgical drainage of the infected collection. Similarly, the application of this method is dubious in case of a widespread infection with non-defined boundaries; furthermore, it is contraindicated in case of a necrotizing infection.

The benefit of the intermittently performed irrigation technique is the possibility to affect the whole infected area surface if only the irrigated and refilled volume of antiseptic solution corresponds with the cavity volume.

Applying antiseptics for topical use shows an upward trend in the treatment of local surgical infection because of an increasing microbial resistance to antibiotics. Theoretically, any topical aqueous antiseptic could be eligible. A correctly chosen antimicrobial agent at an adequate concentration always makes a fast positive effect that is observed in the present study. Several antiseptics were used in this study. Nowadays, hydrogen peroxide has limited indications in the treatment of infections due to its toxic effect on the regeneration of live tissue but the quality of being lethal to the anaerobic microorganisms and to being able to clean out all gaps from pus and debris was the reason for its application in the study patients. From evidence, it is important to underline that the instillation of hydrogen peroxide is painful and for that an injected volume should be less than the cavity volume and must be individualized for each patient. As the main task of a 3% hydrogen peroxide solution is to clean out the cavity from pus and debris, it is enough to apply it only once during the first irrigation.

According to evidence-based World Health Organization guidelines, it is suggested to consider the irrigation of clean or clean-contaminated incisional wounds using an aqueous povidone-iodine solution before closure [10], but preventing and treating concentrations differ. The data on toxic concentration of povidone-iodine are confusing. Gerald Müller et al. [11] demonstrate that a nearly 0.5 % povidone-iodine concentration after a 30-minute exposure has a toxic effect on regenerating cells allowing only 50 % survival of murine fibroblasts. On the other hand, a concentration of 0.5–5% of povidone-iodine is safely used in clinical practice [12].

The current study has some limitations.

In the demonstrated cases, the aspirated collections were relatively small in volume. For that reason there is a lack of evidence as to what extent of the collection volume this approach could be effective and safe. In any event, the procedure must be cancelled if there is no positive effect in 24 h.

The dosage and regimen of the used antiseptics are not validated; therefore, the best protocol in terms of efficacy and safety is still under debate.

## 5 Conclusion

An effective mesh salvaging conservative treatment is possible when the infection manifests itself as an infected fluid collection surrounding the monofilament, large-pore polypropylene mesh and should be the first-line option. Open surgical approach should be reserved as a second-line option if the first fails.

## Conflicts of interest

Nothing to disclose.

## Sources of funding

This research did not receive any specific grant from funding agencies in the public, commercial, or not-for-profit sectors.

## Ethical approval

This study is exempt from ethical approval in our institution

## Consent

Written informed consent was not obtained from the patient 1. The head of our medical team has taken responsibility that exhaustive attempts have been made to contact the family and that the paper has been sufficiently anonymised not to cause harm to the patient or their family. A copy of a signed document stating this is available for review by the Editor-in-Chief of this journal on request. Written informed consent from the patient 2 and 3 was given by themselves personally for publication of their case report and accompanying images. Copies of the written consents are available for review by the Editor-in-Chief of this journal on request.

## Author contribution

Arnolds Jezupovs : Study concept, literature review, writing the paper.

## Registration of research studies

Researchregistry 5445.

## Guarantor

Arnolds Jezupovs Head of Surgical infections clinic

## Provenance and peer review

Not commissioned, externally peer-reviewed.

## CRediT authorship contribution statement

**Arnolds Jezupovs:** Conceptualization, Investigation, Writing - original draft.

## References

[1] Arnold M., Kao A., Gbozah K. (2018). Optimal management of mesh infection: evidence and treatment options. Int. J. Abdom. Wall Hernia Surg..

[2] Sanchez V.M., Abi-Haidar Y.E., Itani K.M.F. (2011). Mesh infection in ventral incisional hernia repair: incidence, contributing factors, and treatment. Surg. Infect. (Larchmt).

[3] Stremitzer S., Bachleitner-Hofmann T., Gradl B., Gruenbeck M., Bachleitner-Hofmann B., Mittlboeck M., Bergmann M. (2010). Mesh graft infection following abdominal hernia repair: risk factor evaluation and strategies of mesh graft preservation. A retrospective analysis of 476 operations. World J. Surg..

[4] Nobaek S., Rogmark P., Petersson U. (2017). Negative pressure wound therapy for treatment of mesh infection after abdominal surgery: long-term results and patient-reported outcome. Scand. J. Surg..

[5] Trunzo J.A., Ponsky J.L., Jin J., Williams C.P., Rosen M.J. (2009). A novel approach for salvaging infected prosthetic mesh after ventral hernia repair. Hernia.

[6] Alston D., Parnell S., Hoonjan B., Sebastian A., Howard A. (2013). Conservative management of an infected laparoscopic hernia mesh: a case study. Int. J. Surg. Case Rep..

[7] Agha R.A., Borrelli M.R., Farwana R., Koshy K., Fowler A.J., Orgill D.P., SCARE Group (2018). The PROCESS 2018 statement: updating consensus preferred reporting of case series in surgery (PROCESS) guidelines. Int. J. Surg..

[8] Bueno-Lledó J., Torregrosa-Gallud A., Sala-Hernandez A. (2017). Predictors of mesh infection and explantation after abdominal wall hernia repair. Am. J. Surg..

[9] Warren J., Desai S.S., Boswell N.D., Hancock B.H., Abbad H., Ewing J.A., Carbonell A.M., Cobb W.S. (2020). Safety and efficacy of synthetic mesh for ventral hernia repair in a contaminated field. J. Am. Coll. Surg..

[10] Allegranzi B., Bischoff P., de Jonge S. (2016). New WHO recommendations on preoperative measures for surgical site infection prevention: an evidence-based global perspective. Lancet Infect. Dis..

[11] Muller G., Kramer A. (2008). Biocompatibility index of antiseptic agents by parallel assessment of antimicrobial activity and cellular cytotoxicity. J. Antimicrob. Chemother..

[12] Buddensick T.J., Cunningham S.C., Kamangar F. (2012). Updated literature on povidone-iodine for control of surgical site infections. Ann. Surg..

